# How myopia develops

**Published:** 2019-05-13

**Authors:** Priya Morjaria

**Affiliations:** 1 Research Fellow: Department of Clincial Research, London School of Hygiene and Tropical Medicine, International Centre for Eye Health, London, UK.

**Myopia is the result of abnormal elongation of the eye, so that light focuses in front of the retina rather than on its surface.**

**Figure:**
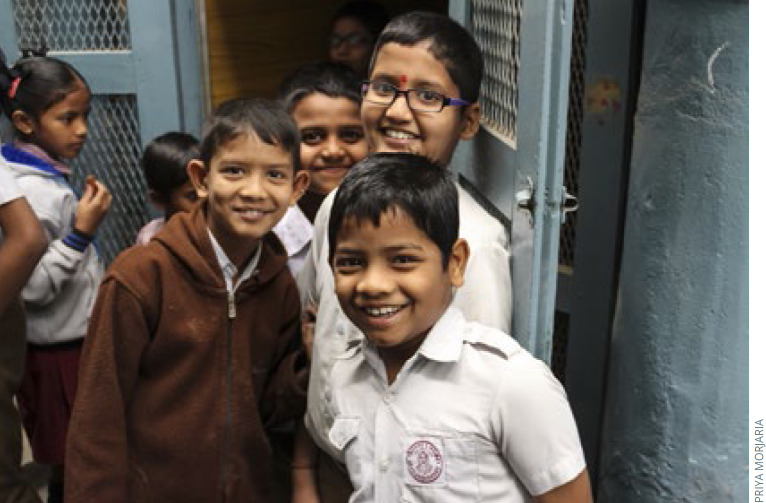
A baby's eyes will grow until they reach a normal length, with no refractive error. In children with myopia, the eye continues to grow. INDIA

Myopia, otherwise known as near- or short-sightedness, is a common type of refractive error. In someone without a refractive error, light rays entering the eye meet and focus on the surface of the retina: the light-sensitive tissue at the back of the eye (Figure 1B). In someone with myopia (Figure 1C), the light focuses at a point in front of the retina, which means that near objects are seen clearly, but objects further away are blurry. This can happen when the cornea is curved too much (is too ‘steep’) or the eye is too long.

Human babies are born long-sighted (hyperopic): distant objects are clear, but near objects are out of focus. This is because they have shorter eyes, so light focuses behind the retina instead of on it (Figure 1A). During the first few years of life, the eyes grow until they reach the expected length, when vision is normal (or emmetropic, see Figure 1B), without any refractive error. In most children, the eye remains like this for life. In some children, the eyes continue to grow and the child develops myopia (Figure 1C).

The eye tends to grow most rapidly during childhood and much slower in adolescence, when physical growth slows down. Children who developed myopia early (at 6–8 years of age) will have had more years during which their eyes can grow rapidly (and their myopia can progress) than those who develop myopia later, at age 12 onwards, when growth is slower. Younger children are therefore at greater risk of eventually developing high myopia (≤ −5 dioptres (D) of correction). Conversely, postponing the onset of myopia, or slowing down the rate at which myopia progresses, will reduce the likelihood that a child will eventually develop high myopia.

**Figure 1:**
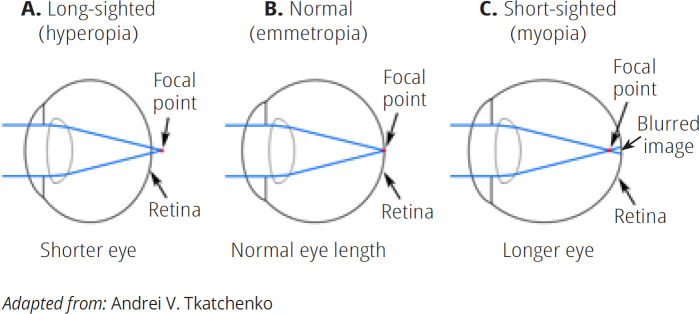
A and B. Normal development of the human eye: from hyperopia at birth (**A**) to emmetropia by early childhood (**B**). Some eyes grow longer and develop myopia (**C**).

High myopia is an irreversible and life-long condition. It can lead to physical changes in the eye, at which point it is referred to as pathologic myopia. In later life, people with high myopia are at greater risk of a range of potentially blinding eye conditions, including retinal detachment, glaucoma, cataract and macular degeneration.

A range of studies suggest that more time spent outdoors is effective at preventing or postponing the onset of myopia, although the precise mechanism for this is not fully understood. Time outdoors is not associated with a slowing of myopia progression in eyes that are already myopic,^1^ but there are optical (p. 19) and pharmacological (p. 21) interventions that have been shown to make a difference.

## What causes myopia?

Several different factors are thought to lead to abnormal elongation of the eyes. Genetic predisposition, environmental factors associated with urbanisation, increased near work and lack of time spent outdoors are all thought to be risk factors associated with myopia. When they act together, the risk increases.^2^,^3^

Many genes are associated with myopia, each affecting a different part of the pathways which influence eye growth. Although a gene location for high myopia has been identified, there are no conclusions about a possible gene location for moderate levels of myopia. The relationship between genetic, optical and environmental factors is difficult to disentangle and remains unclear.
